# Human-Centered Design of an mHealth Tool for Optimizing HIV Index Testing in Wartime Ukraine: Formative Research Case Study

**DOI:** 10.2196/66132

**Published:** 2025-01-30

**Authors:** Nancy Puttkammer, Elizabeth Dunbar, Myroslava Germanovych, Mariia Rosol, Matthew Golden, Anna Hubashova, Vladyslav Fedorchenko, Larisa Hetman, Liudmyla Legkostup, Jan Flowers, Olena Nesterova

**Affiliations:** 1 Digital Initiatives Group at I-TECH Department of Global Health University of Washington Seattle, WA United States; 2 Department of Human Centered Design and Engineering College of Engineering University of Washington Seattle, WA United States; 3 The State Institution «Public Health Center of the Ministry of Health of Ukraine» Kyiv Ukraine; 4 Department of Medicine Division of Allergy and Infectious Diseases University of Washington Seattle, WA United States; 5 STD Control Program Public Health Seattle King County Seattle, WA United States; 6 Biobehavioral Nursing and Health Informatics Department School of Nursing University of Washington Seattle, WA United States

**Keywords:** human-centered design, mobile health, mHealth, Ukraine, HIV testing, war and humanitarian settings

## Abstract

**Background:**

Assisted partner services (APSs; sometimes called index testing) are now being brought to scale as a high-yield HIV testing strategy in many nations. However, the success of APSs is often hampered by low levels of partner elicitation. The Computer-Assisted Self-Interview (CASI)–Plus study sought to develop and test a mobile health (mHealth) tool to increase the elicitation of sexual and needle-sharing partners among persons with newly diagnosed HIV. CASI-Plus provides client-facing information on APS methods and uses a standardized, self-guided questionnaire with nonjudgmental language for clients to list partners who would benefit from HIV testing. The tool also enables health care workers (HCWs) to see summarized data to facilitate partner tracking.

**Objective:**

The formative research phase of the CASI-Plus study aimed to gather client and HCW input on the design of the CASI-Plus tool to ensure its acceptability, feasibility, and usability.

**Methods:**

This study gathered input to prioritize features and tested the usability of CASI-Plus with HCWs and clients receiving HIV services in public health clinics in wartime Ukraine. The CASI-Plus study’s formative phase, carried out from May 2023 to July 2024, adapted human-centered design (HCD) methods grounded in principles of empathy, iteration, and creative ideation. The study involved 3 steps: *formative HCD*, including in-depth individual interviews with clients, such as men who have sex with men and people who inject drugs, and internet-based design workshops with HCWs from rural and urban HIV clinics in Chernihiv and Dnipro; *software platform assessment and heuristic evaluation*, including assessment of open-source mHealth platforms against CASI-Plus requirements, prototype development, and testing of the REDCap (Research Electronic Data Capture) prototype based on usability heuristics; and *usability walk-throughs*, including simulated cases with HCWs and clients.

**Results:**

The formative phase of the CASI-Plus study included in-depth individual interviews with 10 clients and 3 workshops with 22 HCWs. This study demonstrated how simplified HCD methods, adapted to the wartime context, gathered rich input on prioritized features and tool design. The CASI-Plus design reflected features that are both culturally sensitive and in alignment with the constraints of Ukraine’s wartime setting. Prioritized features included information about the benefits of HIV index testing; a nonjudgmental, self-guided questionnaire to report partners; client stories; and bright images to accompany the text. Two-way SMS text messaging between clients and HCWs was deemed impractical based on risks of privacy breaches, national patient privacy regulations, and HCW workload.

**Conclusions:**

It was feasible to conduct HCD research in Ukraine in a wartime setting. The CASI-Plus mHealth tool was acceptable to both HCWs and clients. The next step for this research is a randomized clinical trial of the effect of the REDCap-based CASI-Plus tool on the number of partners named and the rate of partners completing HIV testing.

## Introduction

### Background

In December 2016, the World Health Organization recommended scale-up of HIV assisted partner services (APSs) as a strategy to increase the identification and testing of partners of persons with HIV [1,2]. APSs offer persons with HIV assistance to confidentially notify their sexual and needle-sharing partners of their exposure and link the partners to testing and treatment. Randomized controlled trials have shown that APSs increase HIV testing and case finding [3-6] and are cost-effective [7,8]. APSs are scalable in routine practice, but routine APS programs have typically demonstrated lower HIV case finding than was observed in randomized controlled trials [9-12]. At least in part, the low case finding observed with APSs in most settings reflects the small number of sexual and needle-sharing partners named by index cases. As of 2020, persons with HIV receiving APSs in Ukraine named an average of only 1.14 partners despite training of APS program personnel that emphasized the need to elicit more than 1 partner for each index client [13].

Computer-Assisted Self-Interview (CASI)–Plus seeks to improve partner elicitation and testing through a mobile health (mHealth) client engagement tool using a CASI. CASI-Plus supports the initial APS encounter by providing information on how APSs work, eliciting names of all sexual or injection partners, screening for risk of intimate partner violence, and planning for partner notification. It also facilitates case management at the point of partner testing and via repeat follow-up surveys to assess for barriers to notification, interest in provider assistance, and occurrence of intimate partner violence. In surveys of sexual behavior across diverse settings, the computer-assisted self-interview approach reduced social desirability bias compared with in-person interviews [14-17]. However, the computer-assisted self-interview approach has not yet been used in APS programs in low- or middle-income countries. The CASI-Plus study sought to assess whether the tool improves partner elicitation and HIV testing in a routine APS program operating at scale, with the goal of increasing the number of persons with HIV who know their status and are linked to either HIV pre-exposure prophylaxis or HIV antiretroviral therapy services. The Russian Federation’s full-scale invasion and ongoing war in Ukraine has underscored the need for simple and scalable interventions to support the control of the HIV epidemic in a disrupted health care system. CASI-Plus offers the potential to improve APS partner elicitation and HIV case finding in wartime Ukraine, as well as in many other settings where routine APS programs face challenges with high-fidelity APS implementation and low contact (partner) elicitation.

### Objectives

For the CASI-Plus study, we first carried out formative research with health care workers (HCWs) and clients from a human-centered design (HCD) perspective. As applied to mHealth tools, HCD is a pragmatic research orientation that seeks to overcome barriers to usability and integration with workflows that arise when tool designers ignore the sociotechnical complexity of health care systems [18,19]. HCD principles and methods emphasize engaging with mHealth users, starting with problem definition before solution design; supporting divergence and convergence of ideas through the design process; using participatory co-design techniques, prototyping, and iteration; and applying values of creativity, empathy, tolerance for ambiguity, and willingness to fail [18,20-22]. There are few research studies or frameworks discussing the application of HCD perspectives in mHealth projects in wartime and humanitarian contexts. The participatory engagement and time required to carry out HCD-oriented research may be challenging in wartime contexts [23], and more scholarship is needed on realistic approaches to HCD in such contexts. During the CASI-Plus formative research phase, we adapted HCD methods to Ukraine’s wartime context. The aims of our formative research were to (1) describe how HCW and client perspectives and the wartime context of Ukraine shaped the design of the CASI-Plus mHealth tool and (2) identify HCD methods and principles for effective formative mHealth research in a wartime setting.

## Methods

### Study Design

The formative phase of the CASI-Plus study (ClinicalTrials.gov NCT05826977) supported the design and software adaptation of an mHealth tool for use in Ukraine’s APS program. During the first step of the formative research, we conducted internet-based workshops with HCWs from 3 health facilities, as well as in-depth interviews with clients enrolled in HIV care at 1 health facility. During the second step, we developed the CASI-Plus tool, rated its usability based on common usability heuristics or “rules of thumb,” and made improvements to the prototype. During the third step, we obtained feedback on the CASI-Plus tool prototype through simulated use of the tool by health workers and clients to further adapt our design. Table 1 presents an overview of the activities across the 3 steps of our formative research.

**Table 1:** Summary of the Computer-Assisted Self-Interview (CASI)–Plus formative research design—participants, methods, and activities for research steps 1 to 3 in wartime Ukraine (May 2023-July 2024).

	Step 1: formative design workshops and interviews —elicit and prioritize CASI-Plus features			Step 2: heuristic evaluation —critique platforms and prototype		Step 3: simulated walk-throughs—end user prototype testing	
	Health care workers	HIV care clients	Study team		Health care workers		HIV care clients
Method	Design workshops with focus group	In-depth individual interviews	Compare tools and review CASI-Plus usability		Review of prototype and focus group		Review of prototype and individual interviews
Activities	Preworkshop exercise Critique ideas for CASI-Plus features Feature prioritization game Group synthesis and feedback	Discuss current APS^a^ barriers Critique CASI-Plus mock-up Review client script	Compare 5 open-source platforms with CASI-Plus requirements from step 1 Critique CASI-Plus REDCap^b^ prototype using usability best practices		Add 5 fictional clients to prototype and test features Discuss feedback with the group		5 current HIV care clients walked through the client portion of CASI-Plus Gather feedback

^a^APS: assisted partner service.

^b^REDCap: Research Electronic Data Capture (Vanderbilt University).

### Ukrainian Setting and War

Ukraine is an Eastern European nation that regained its independence from the Soviet Union in 1991. On February 24, 2022, the Russian Federation invaded Ukraine, inflicting the largest military assault in Europe since World War II. Of a prewar population of 41.6 million, an estimated 7.5 million (18%) fled the country as refugees, 5.9 million (14.2%) were internally displaced, 1.6 to 2.8 million were forcefully deported to Russia, and >10,000 civilians were killed [24-26]. As of mid-2023, when this study was conducted, the population was estimated to be 41 million, with 12.4% being internally displaced persons and 11.6% being refugees who had returned to their homes [27]. War crimes have included direct attacks on health care facilities, resulting in damage to >1000 facilities, complete destruction of >200 facilities, and deaths of >100 HCWs (with approximately one-third killed while working) [28-31]. Our study sites included health facilities in the cities of Chernihiv and Dnipro. Chernihiv is in northern Ukraine and was occupied by Russian forces from March 2022 to April 2022, leading to widespread destruction and trauma [32]. Dnipro is a leading industrial city located in southeastern Ukraine that has served as a hub for wartime logistics and humanitarian aid for the eastern and southern regions most affected by war and occupation.

### HIV in Ukraine

Ukraine has a significant HIV burden, with an estimated 245,000 persons with HIV living in the country in early 2022, of whom roughly 79% knew their HIV status [28]. In 2019, the Ukraine Ministry of Health began scaling APSs, reaching 7533 index clients and 5038 newly tested partners in 2020 [13]. Clients who are newly diagnosed with HIV, as well as clients who were previously diagnosed but who experienced interruptions in HIV treatment or other conditions leading to having an unsuppressed HIV viral load, are invited to participate in APSs on a voluntary basis. Index clients are asked to name all recent sexual and needle-sharing partners as well as biological children to indicate whether these partners have recently been tested for HIV and to select a notification method for each partner. Health workers then follow up over time with the index clients to ensure that each partner is notified and provided information about how to get tested for HIV. Persons who inject drugs and men who have sex with men have high HIV prevalence (20.9% and 3.9%, respectively) and are estimated to account for 29.9% and 2.9% of all persons with HIV in Ukraine [33-36]. However, these key populations appear underrepresented in APSs as persons who inject drugs made up only 14.9% and men who have sex with men made up only 1.8% of APS index clients in 2020 [13,37,38]. An additional barrier to epidemic control in Ukraine was that approximately one-third of named partners with unknown status (32.4%) in the APS program did not complete HIV testing within 60 days [13].

In the wartime context, HIV programs have been stretched, and conditions have favored increased HIV transmission [39]. HIV testing declined by 36% in 2022 compared to 2021 before rebounding to prewar levels in 2023 [28]. The Ukraine Ministry of Health estimated that reduced coverage of HIV testing services, together with wartime occupation and population displacement, contributed to a 13% increase in HIV incidence in 2023 compared to 2022 [28]. In the wartime context, APSs remain an expected part of the basic package of HIV services. However, their prioritization during the war given other pressing needs has not been thoroughly assessed, including in the context of our study.

### HCD Methods for the Design of CASI-Plus

We sought to design CASI-Plus as a tool to increase partner elicitation and testing that would be both tailored to Ukraine’s specific wartime context and generalizable to APS programs worldwide. We selected HCD methods given their utility in leveraging end-user expertise and taking a system thinking approach while designing this health care innovation [19,22,40]. Melles et al [18] discuss the importance of system thinking regarding how an innovation interacts across 3 levels: micro (eg, using tools to perform tasks), meso (eg, health workers as a part of teams that coordinate to deliver care), and macro (eg, health workers and clients as a part of organizations and societies). With HCWs, our inquiry focused on eliciting task-based features (micro level), as well as how to integrate a tool within their health care delivery workflow (meso level) and how to tailor the design for the Ukrainian cultural context and wartime environment (macro level). For index clients, our study focused on considerations to ensure that the tool was accessible (micro), aligned with patient health care experiences (meso), and aligned with client values (macro).

Our original study, conceived before the full-scale invasion of Ukraine in 2022, proposed in-person, participatory HCD workshops and interviews with HCWs and clients led jointly by American and Ukrainian investigators. However, in the wartime context, we needed to reconsider our HCD methods based on disrupted ground transportation, safety of Public Health Center (PHC) of the Ministry of Health of Ukraine and University of Washington study team members to travel to the study sites, understaffing at the study sites, and unpredictable client demand for health care services due to mass population displacement and military mobilization. Table 1 provides an overview of HCD activities across 3 steps, whereas Table 2 provides a more detailed description of the internet-based, tele-conferencing sessions with HCWs and in-person engagements with index clients and how the HCD methods were adapted to the wartime context.

**Table 2:** Detailed description of human-centered design (HCD) methods used in the Computer-Assisted Self-Interview (CASI)–Plus study, including HCD principles and adaptations to Ukraine’s wartime context (May 2023-July 2024).

Step, participants, and activity		Description	HCD principles	Adaptations to wartime context
**Step 1—HCWs^a^**				
	Individual preworkshop exercise 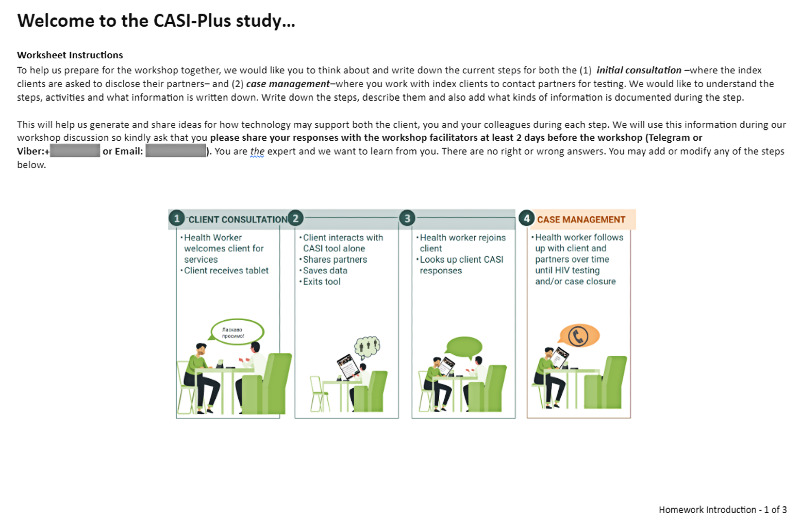 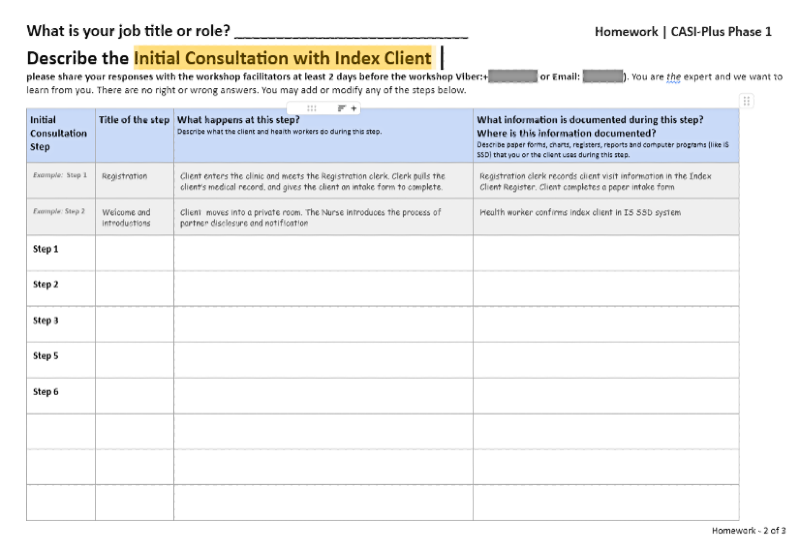	HCWs created an ordered list of the steps of APSs^b^ and described care activities, who was involved, and the data collected. HCWs were reminded of their expertise and that there were no right or wrong answers. The study team consolidated individual workflow maps to produce a consensus version for display during the workshop session.	Empowering end users as experts—presession exercises can prime users’ engagement and allow them to reflect independently and without the social pressures of the workshop [41,42]; crowdsource responses from multiple participants—this helps consolidate core steps, which is critical to understand context and activities that the tool could support [18,43]	Reduced the duration of the design workshop and allowed participants to complete when they had availability (thereby reducing burden). Allowed the study team to consolidate information for review at the workshop. Included explicit discussion of how war has impacted clients and services.
	Critique ideas for CASI-Plus features 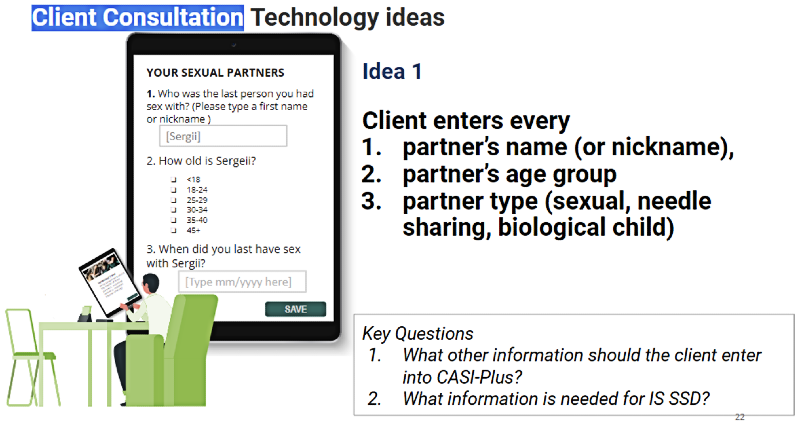 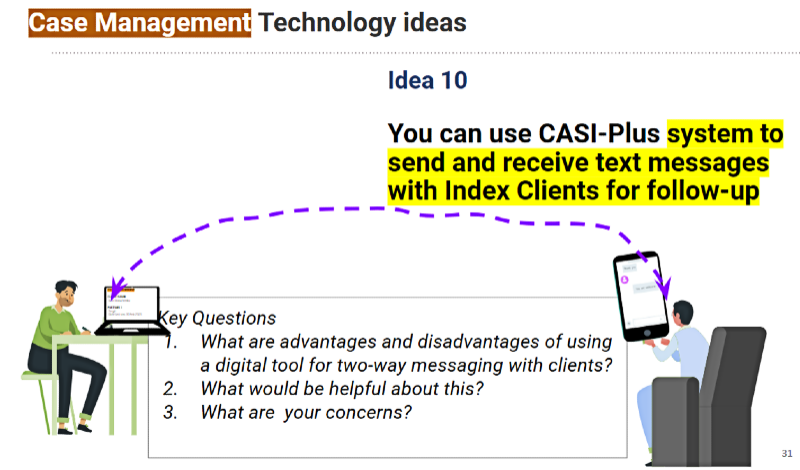	Reviewed mock-ups of 12 ideas for CASI-Plus features. Each mock-up contained key discussion question prompts (eg, “what are the advantages of this feature?” and “what are your concerns?”). Invited participants to share additional feature ideas and describe ideal feature designs that would be most useful.	Ideation and creativity [20]; co-design process supported design and validation of a solution based first on diverging perspectives and then on converging perspectives	Conducted the workshop via videoconference (Zoom [Zoom Video Communications]) where participants joined individually to minimize safety risks and the inconvenience of in-person meetings. Web-based tools for ideation and creative work (eg, Figma) were deemed too complex for HCWs to learn and use, so the study team relied on slides and discussion. Visual mock-ups supported reactions and discussions and stimulated creative thinking.
	Feature prioritization game 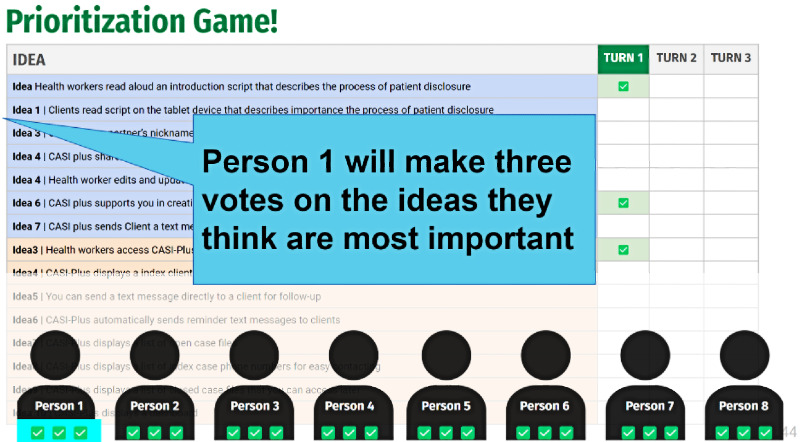	HCWs completed 3 rounds of voting on priority features, with each HCW having 3 votes per round. Between rounds of voting, HCWs discussed why they prioritized specific features. One group received 3 extra votes for the final round when they did not change choices between the first and second round.	The prioritization game helped focus the feature set for health worker top preferences. The prioritization game was adapted to voting on features rather than a money-based game of purchasing options by the study team per recommendations that voting would resonate more with participants.	Used verbal roll call for voting along with marking each vote on the screen to avoid the complexity of web-based voting tools. HCWs were engaged in exercises, discussion, and explaining their choices.
	Group synthesis and feedback	Semistructured focus group discussions on how CASI-Plus would impact workflow and what barriers it would and would not address	Consider user needs and experiences [43]; system perspective	HCWs required extra time to familiarize themselves with and practice using the tool after the internet-based orientation.
**Step 1—clients**				
	Discuss current APS barriers 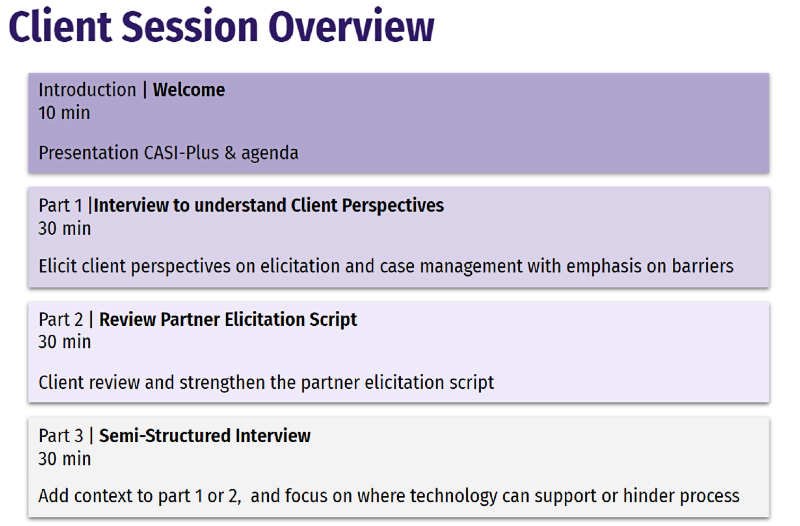	Semistructured questions on barriers to naming partners, notifying partners, using APSs, and partners completing testing. Reflections on what works well and what can be improved in APSs.	Empathy [20]	IDI^c^ questions asked about barriers in the wartime context.
	Critique CASI-Plus features and review client script 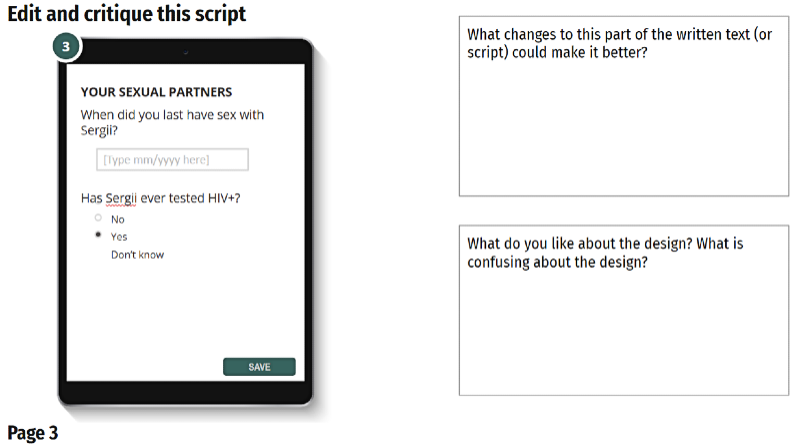	Semistructured questions on CASI-Plus features, including opinions on 2-way texting and reflections on what barriers it would and would not address. Clients reviewed 13 cards with visual mock-ups of the client-facing screens of the CASI-Plus tool. They shared reactions, including things that were confusing or inappropriate. They provided alternative suggestions for visual design.	Co-design; creativity	A single round of design feedback was conducted to minimize burden on client and researcher time in wartime context.
**Step 2—study team**				
	Heuristic evaluation to critique platforms and prototype 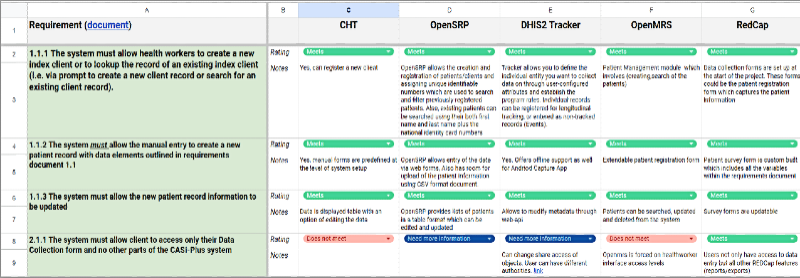 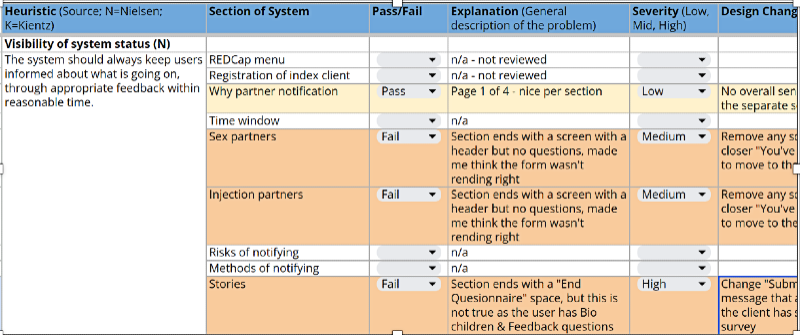	Evaluated 5 open-source software platforms against the CASI-Plus requirements developed by the study team and refined during step 1. Conducted heuristic evaluation (usability “rules of thumb”).	Heuristic evaluations are common in HCD in which study team members compare a tool against heuristics to fix common usability issues [44,45].	Ukrainian and American study team members independently and asynchronously completed this activity and documented the results in a spreadsheet to minimize the need for synchronous web-based meetings.
**Step 3—HCWs**				
	Usability walk-throughs 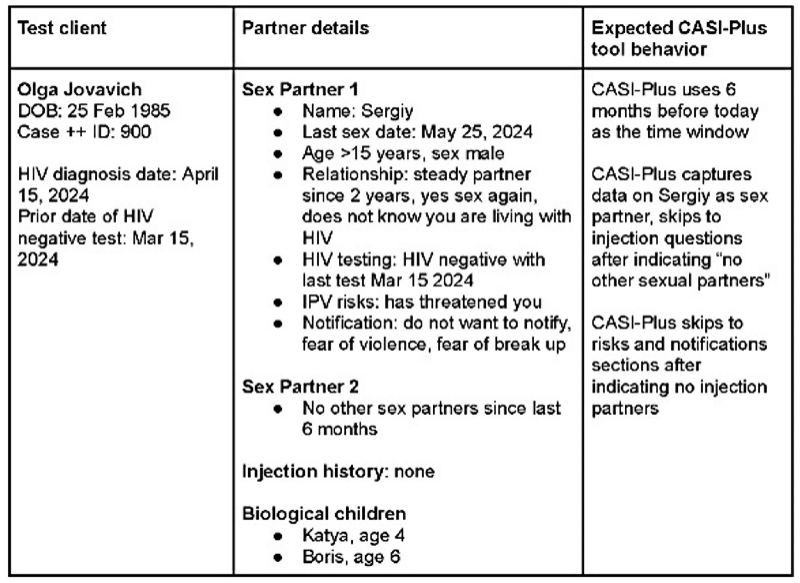	Provided “test scripts” for 5 fictional clients for HCWs to enter data and a template for documenting issues and recommendations.	Consider user needs and experiences [43].	The study team met via videoconference with HCWs to debrief their walk-throughs.
**Step 3—clients**				
	Usability walk-throughs 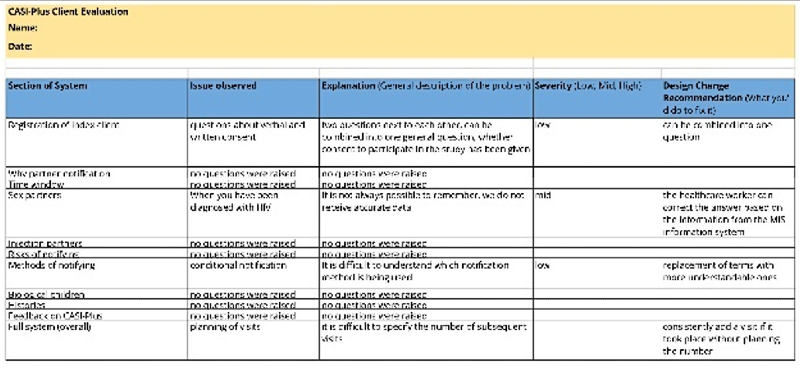	A total of 5 clients participating in APSs tested CASI-Plus using a “talk aloud method.” HCWs documented issues and recommendations in a structured template.	Consider user needs and experiences [43].	Clients rejected being video recorded when piloting the use of the tool.

^a^HCW: health care worker.

^b^APS: assisted partner service.

^c^IDI: in-depth interview.

### Step 1: Formative HCD (May 2023-July 2023)

During step 1, with HCWs, we asked participants to (1) complete a preworkshop exercise to describe current APSs, including which personnel and what data collection or data management tools were involved; (2) discuss the current state of APSs, including barriers to partner notification and testing; (3) review 12 ideas for CASI-Plus features; and (4) prioritize features during a voting game in which participants selected top features, discussed their choices, and repeated 3 rounds of voting to allow them to change their priorities. We also carried out individual in-depth interviews with clients in which we asked them to (1) discuss current barriers to partner notification and testing and (2) critique mock-ups showing the client-facing screens and CASI-Plus tool design.

HCW workshop participants included a purposive sample of site managers, infectious disease physicians, nurses, psychologists, and social workers who deliver APSs and HIV care services. The first 2 HCW workshops included HCWs from the regional AIDS centers at hospitals in Chernihiv and Dnipro. The third workshop included HCWs from smaller rural primary care clinics that operate as subsidiary locations for the Chernihiv site. Because of safety concerns with travel of researchers to the study sites, the 3 HCW workshops were held via Zoom videoconference (Zoom Video Communications), with voluntary video and audio recording of the sessions. In total, 2 Ukrainian researchers facilitated the videoconference workshops using a facilitator’s manual and slide decks. The English-speaking study team members listened in silently with synchronous translation to follow the proceedings and observe group dynamics. Before the workshops, participants were given an individual preworkshop assignment to describe APS consultation and case management activities. Between the first and second workshops, given the HCD approach, the study team iterated on potential CASI-Plus features to reflect feedback and better tailor ideas for the subsequent 2 HCW workshops.

For index clients, the study team held 30- to 45-minute in-person interviews at a single site because we could not assume that clients had access to the internet or familiarity with videoconferencing methods. We also reduced the number of client touch points during step 1 from 2 to 1 session to reduce participant burden during wartime. Clients were recruited from the Chernihiv oblast AIDS centers using a convenience sample of clients coming for routine medical visits. Inclusion criteria were (1) being aged ≥18 years and (2) having participated in APSs within the previous 12 months at the study clinic. Because we were particularly interested in the perspectives of clients from vulnerable, stigmatized groups, including men who have sex with men and persons who inject drugs, study personnel worked with HCWs to ensure that clients from these groups were referred to the study.

All workshops and client interviews were conducted in the state language, Ukrainian.

### Step 2: Software Platform Assessment and Heuristic Evaluation (October 2023-May 2024)

During step 2, the study team evaluated 5 open-source software platforms against the CASI-Plus functional and technical requirements. The study team developed the requirements based on work in a preliminary study and updated this based on feedback from step 1. We then adapted the selected platform (REDCap [Research Electronic Data Capture; Vanderbilt University]) [46,47] to become the CASI-Plus tool and completed heuristic evaluation to assess usability. Each heuristic, or rule of thumb, for what makes for a pleasing and usable electronic tool was drawn from frameworks by Nielsen [44] and Kientz et al [45] using concepts such as “helps users recognize and recover from errors or relies on recognition rather than recall.” We created a matrix tool for rating usability according to each heuristic applied to each section of the CASI-Plus tool, with coding of each issue as having low, medium, or high impact on usability (Multimedia Appendix 1). Study team members then conducted walk-throughs of the tool with 5 fictional client “test scripts,” rating each part of the CASI-Plus tool as passing or failing each heuristic. We collated all issues and addressed all modifiable high- and medium-impact issues through a round of improvements to the tool.

### Step 3: Usability Walk-Throughs (July 2024)

Step 3 involved HCWs and clients conducting simulated walk-throughs of CASI-Plus at the Chernihiv study site using a live test version of the tool with the Android tablets procured for use of CASI-Plus at study sites. HCWs used the 5 fictional client test scripts, where they registered clients in the live version of CASI-Plus, entered client data, viewed the summary results, and edited client data as needed. After these walk-throughs, the PHC team held a focus group to gather feedback on navigation, system performance, and concerns using CASI-Plus in practice. In addition, the study coordinator completed walk-throughs with 5 actual clients, again using the live version of the tool and the tablets, followed by brief individual interviews to gather feedback, learn whether they would recommend the tool’s use by others, and share suggested changes to the tool. Participants received similar incentives as in step 1.

### Data Analysis

Ukrainian study team members created English-language rapid debrief reports for the data collected in step 1 using a template that followed the semistructured discussion guides to support rapid qualitative analysis [48-51]. Rapid qualitative analysis enabled actionable insights to emerge from each HCW workshop so that we could iterate on the ideas and visuals to consider input from each session. Later, full written transcripts were created and translated into English for additional analysis to reveal themes related to Ukraine’s wartime and social context and inform the tool design. One analyst (NP) developed an inductive thematic codebook after an initial read-through of the transcripts and then applied the codebook to each transcript using the Dedoose software (SocioCultural Research Consultants) [52]. Data from steps 2 and 3 were tracked in spreadsheets and used in conjunction with the software requirements and design documents to guide modifications to the software prototype.

### Ethical Considerations

The protocol for the CASI-Plus study was reviewed and approved by the University of Washington Human Subjects Division (00015808) and the institutional review board for the PHC (222; August 12, 2022). All participants provided written informed consent using a Ukrainian-language consent process and form. Health workers and clients were each offered an incentive of US $10 for participation in the CASI-Plus formative research. All data collected from study participants were deidentified before analysis and stored in secure, password-protected electronic storage locations.

## Results

### Overview

There were 22 HCWs and 10 clients participating in step 1 data collection (Table 3). All HCW participants (22/22, 100%) were female, most (13/22, 59%) were infectious disease or other physicians, and most (18/22, 82%) had provided HIV services for ≥5 years. Clients interviewed were mostly male (8/10, 80%) and included both persons who inject drugs (6/10, 60%) and men who have sex with men (4/10, 40%). Most clients interviewed (6/10, 60%) were aged 35 to 44 years.

**Table 3:** Study participants for step 1 of the Computer-Assisted Self-Interview–Plus formative research in wartime Ukraine (May 2023-July 2023).

Characteristic			Participants, n (%)
**Health workers (n=22 individuals in 3 workshops)**			
	**Location of work**		
		Oblast AIDS center	15 (68)
		Peripheral health center	7 (32)
	**Sex**		
		Female	22 (100)
		Male	0
	**Professional cadre**		
		Infectious disease physician	11 (50)
		Nurse	7 (32)
		Other physician	2 (9)
		Psychologist	1 (5)
		Social worker	1 (5)
	**Time working in HIV program (y)**		
		>10	8 (36)
		5-10	10 (45)
		2-5	4 (18)
	**Age group (y)**		
		<35	4 (18)
		35-44	8 (36)
		≥45	10 (45)
**Clients (n=10 in-depth interviews)**			
	**Sex**		
		Male	8 (80)
		Female	2 (20)
	**Key population group**		
		Persons who inject drugs	6 (60)
		Men who have sex with men	4 (40)
	**Age group (y)**		
		<35	2 (20)
		35-44	6 (60)
		≥45	2 (20)

### Step 1: Formative HCD

#### APS Workflow

HCWs across the 3 workshops identified common, standard steps for the APS workflow, matching steps in the index client journey through initial and follow-up visits (Multimedia Appendix 2). The workflow activity resulted in a consensus on a standardized workflow that harmonized perspectives of HCWs within and across study sites. Noting that APS clients are newly engaged in care, HCWs discussed how some clients needed to build trust in the health care team before disclosing information about their partners, which underscored that CASI-Plus should be designed for longitudinal partner elicitation rather than elicitation at the initial APS enrollment visit. HCWs at one site reported eliciting information about household members and friends (in addition to HIV-exposed partners and biological children); however, the PHC researchers determined that “social network testing” was not part of the standard APS model and should not be a focus of the tool.

#### APS Barriers and Effects of Wartime

HCWs and clients noted barriers to APSs and challenges of wartime; Table 4 summarizes these barriers and challenges with illustrative quotes. Barriers to partner notification included fear of blame, rejection, family dissolution, and aggressive reactions or abuse by partners, as well as clients’ inability to come to terms with their HIV diagnosis. Both HCWs and clients discussed stigma and discrimination in health care by family physicians and other medical providers as being a barrier to HIV testing. Both groups also cited practical barriers to partner notification and testing, such as difficulty traveling to health facilities due to disrupted transportation networks, difficulty tracking partners due to widespread population displacement, and hesitancy to accept calls from unknown numbers given the efforts of military recruiters to enlist citizens in the armed forces. HCWs at the Chernihiv site explained that they did no HIV testing for 2 months while under Russian occupation in 2022. They estimated that HIV testing levels had rebounded to approximately 75% of the level in 2019, before the war and the COVID-19 pandemic, while noting that understaffing was an ongoing challenge in delivering APSs.

**Table 4:** Barriers to HIV assisted partner services (APSs) in wartime Ukraine—results from step 1 in-depth client interviews and health worker workshops (May 2023-July 2023).

Barrier	Themes	Illustrative quotes
Lack of acceptance of HIV diagnosis	Denial of HIV test result Anger and blame of others	“To be honest, I didn’t immediately accept it, because I was on my own, I had the Internet at first, I was searching for all possible options why the test could be wrong. Well, I had a feeling that I wanted to turn this diagnosis inside out.” [Client; male; man who has sex with men; aged 32 years] “[A] person is offended that he/she was punished in this way, so they want to punish others, I believe. I had a situation like that...there was a time when I blamed everyone, I wanted to infect the whole world...[A] psychologist worked with me and everything is normal now, touch wood.” [Client; female; person who injects drugs; aged 44 years]
Concern about disclosure of HIV status	Fear of “word getting out” about one’s HIV status Even an anonymous notification by an HCW^a^ could allow a partner to deduce the source of the exposure	“So, you [health worker] are calling and saying: ‘Here, we have been informed...that you had sexual contact during last year.’ The thing is, that we don’t know how many were there, right? But he does. He knows that he was only with me.” [Client; male; man who has sex with men; aged 32 years]
Fear of partners’ reactions	Women and clients with newer partners may have more trouble notifying their partners A client who was a man who has sex with men reported greater difficulty notifying bisexual partners because of fear of blame if wives and children become affected	“[Y]ou’re not, like, the same person anymore. You’re HIV-positive. It’s honestly stressing me out...[A] lot of people kind of say don’t be afraid of this, but I’m already a second-class person.” [Client; male; person who injects drugs; aged 44 years] “[I]f, for example, he was single, yes [I could notify him]. Whatever was to happen let it happen. [But if] he is married, it’s ok if everything is fine. But if not, it would be chaos in the family...I'm afraid of the reaction, so to speak.” [Client; male; man who has sex with men; aged 32 years]
Clients have other priorities	Clients who are persons who inject drugs are generally difficult to engage due to their preoccupation with obtaining drugs or money for drugs if they are actively using drugs Fatalistic wartime attitude Low obligation to notify partner if they do not have an ongoing relationship	“Due to the war, some clients have adopted a fatalistic attitude towards life and do not wish to inform their partners, making an excuse that they will live as long as they live, and what if they die tomorrow from a rocket.” [HCW; female; 8 years of professional experience] “[W]hen...I met my partner just once or twice...we met, he texted me or called me, I answered him, we met, and I don’t even sign this [phone] number. Especially if I didn’t like the partner, then, well, what’s the point?” [Client; male; man who has sex with men; aged 32 years]
Difficulty disclosing partnerships to HCWs due to stigma	Shame to admit to health workers that clients have sex partners who are men who have sex with men or that they have shared needles Social stigma can prevent partners from accessing HIV testing services because they do not want to be seen entering the HIV clinic for testing	“[T]hey do not name their partners in all cases, they say ‘I have no partner,’ and then, after a while, we conduct conversations, we figure out that a partner of this or that client presented to us...It happens after some time, not immediately when a patient has just found out his status, rather after a while.” [HCW; nurse; 8 years of professional experience] “[T]here are some people who are coming here and try to hide the fact that they are using [drugs]...or they may say the opposite, ‘I’m quitting’ or something else...[T]hey will be afraid, if they have to tell the doctor, the doctor will just swear or will be negligent...like: ‘Oh, they’re drug addicts?...[T]hey’re so...annoying,’...so they ignore it and don’t talk about it.” [Client; female; person who injects drugs; aged 43 years]
Ethical concerns	Concern about sharing partner contact details without asking them first (client) Preference for routine HIV testing as part of primary health care, alleviating responsibility for notifying partners (client)	“[P]ersonally, I don’t provide phone numbers to anyone without asking the person if I can. I don’t know if other people do it.” [Client; male; man who has sex with men; aged 39 years]
Practical barriers to partner notification and testing	People may not pay attention to phone calls or messages attempting notification (HCWs and clients) Massive displacement of the population during war creates difficulties tracing and contacting partners Disrupted transportation networks make it difficult for citizens from rural and semiurban areas to access HIV testing	“Due to the war, male clients and male partners are much more hesitant to answer calls from unknown numbers. They fear mobilization and are apprehensive about receiving calls from the military, so very often they don’t pick up or don’t trust strangers on the phone.” [HCW; female; >10 years of professional experience]
Skeletal, overburdened HCW staff is challenged to provide APSs	HCWs have limited bandwidth to track partners over multiple attempts due to workload Too few social workers to help physicians with APSs	“I have 2 social workers here. One works part-time 25 per cent, which is 1 hour and 28 minutes, the other works part-time 75 per cent. A patient comes for ARVs...you need to find out about their sexual partners, you need to ask them about complaints, enter it [data] into the system of socially significant diseases, simultaneously enter it into the NHSU, into e-Health, so that we are paid for this case, write them out a prescription, refer them to tests… we are given 12 minutes per each patient...[Y]ou have to do your best, talk through everything quickly...There is such a race with each patient. And how to do all this, to ask them about all partners...they sit and try to remember, it can only be done properly in the social worker’s office, if they are free...” [HCW; female; unit head; 23 years of professional service]

^a^HCW: health care worker.

#### Perceptions of the CASI-Plus Tool

Both HCWs and clients had an overall favorable perception of the CASI-Plus tool. HCWs and clients both felt that it would help clients discuss their partnerships without shame or embarrassment:

...there are patients who are sometimes ashamed to tell the doctor how many partners they have, because the person lives in a marriage and does not really want to reveal that they have someone anyone else, you now, and when it will be on the tablet, we will not look into their eyes, into their soul, as they say, perhaps the person will then simply open up. Perhaps a person will not name them, but at least they will say the number. As if it will be useful for us.

HCW; female; >10 years of professional experience

One client felt that it would help providers remember to discuss partner testing with each client:

...it’s a great idea with a tablet, why, if the doctor, let’s say, forgets to ask something, then that’s it, I’m gone, I’ll go home and think “[shoot], but I still wanted to talk about my problem, well, I won’t, okay, later.”

Client; female; person who injects drugs; aged 43 years

Of the 10 clients interviewed, all (100%) expressed willingness to use CASI-Plus on a tablet within the clinic, and only 1 (10%) expressed a definitive preference for speaking with an HCW about APSs rather than using CASI-Plus. HCWs expressed concern about the physical security and maintenance of tablet devices and made recommendations for preventing theft.

#### Preferred Features for the CASI-Plus Tool

**Figure 1:**
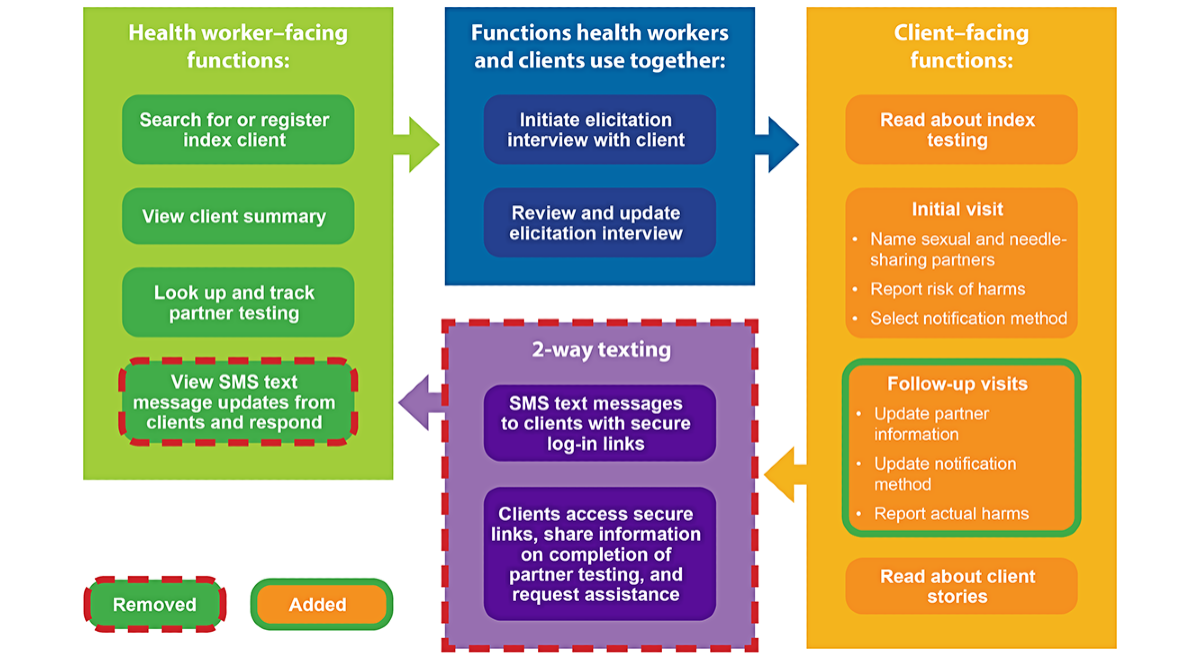
Evolution of the Computer-Assisted Self-Interview–Plus functional design based on step 1 formative research in wartime Ukraine (May 2023-July 2023).

Figure 1 shows the original concept for the CASI-Plus features, input from HCWs and clients about the features, and how we modified the design to address the feedback. The most important changes were (1) dropping 2-way texting with clients and (2) modifying the case management dashboard to focus on person-level information about partner disposition as reported by index clients rather than APS program performance metrics. Multimedia Appendix 2 summarizes feedback and shows how the design evolved across each step of the APS workflow, whereas Multimedia Appendix 3 shows the results of the HCW prioritization voting exercise.

During the 3 HCW workshops, HCWs prioritized features that support the initial APS encounter, including educational messaging about the rationale for APSs and how they work, as well as an initial partner elicitation questionnaire. Some HCWs and clients liked the idea of client stories that shared realistic examples of clients who pursued partner notification and testing. Clients liked the idea of CASI-Plus including 2-way texting because they felt that it would be a convenient way to communicate with their health care team. They felt that encrypted messaging (eg, via the Viber platform) would protect the privacy of communication. However, HCWs were concerned that 2-way texting would add to their workloads even if messaging was partially automated. They were also concerned about operating outside the current national legislative framework, which narrowly limits the collection and use of private information such as names and phone numbers.

**Figure 2:**
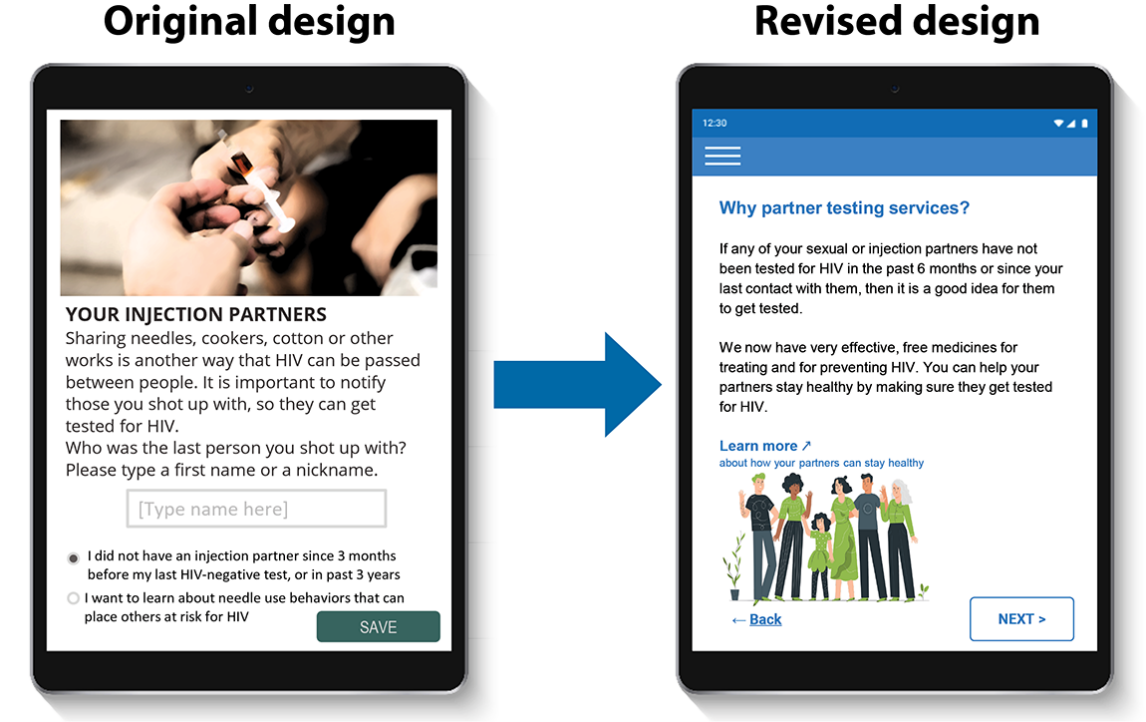
Evolution of the Computer-Assisted Self-Interview–Plus visual design based on step 1 formative research in wartime Ukraine (May 2023-July 2023).

There was less consensus among HCWs about CASI-Plus functionality to support APS case management. HCWs were enthusiastic about having a dashboard display of information that would help them track whether partners had completed HIV testing. However, they did not value a dashboard with performance indicators such as the total number of clients served or the average number of partners named per index client. HCWs said that they could not handle the additional data entry of case updates about contact attempts or partner testing disposition within the tool given their existing workload. They recommended that clients capture partner follow-up data via a self-administered follow-up CASI-Plus questionnaire to be used whenever they returned to the clinic. Clients requested more health promotion information, such as messages normalizing HIV and explaining benefits of HIV pre-exposure prophylaxis and antiretroviral therapy. Design preferences included a large font for readability and a bright and hopeful visual design, with images that could help clients easily follow the text (Figure 2).

### Step 2: Software Platform Assessment and Heuristic Evaluation

An evaluation of 5 widely used open-source technology platforms for digital health resulted in the initial selection and adaptation of OpenSRP (Ona Systems) [53] as the optimal platform to meet the detailed functional and technical requirements developed by the team for CASI-Plus. The other 4 platforms considered were the Community Health Toolkit [54], DHIS2 Tracker [55], OpenMRS [56,57], and REDCap [46,47,58]. However, after building CASI-Plus using OpenSRP, the study encountered potential delays in implementing the tool due to risks posed by the ongoing war. The use of technology in Ukraine, particularly in wartime, required a careful approach to ensure data security and prevent leaks, reflecting a broader state-level policy on protecting personal and medical data. In the context of Russian cyber warfare threats, hosting CASI-Plus on secure, trusted servers within Ukraine became a top priority. Therefore, the study team emphasized the specific operational needs of having a platform with an appropriate license, a Ukrainian-language interface, and reliable technical support. These requirements were critical for aligning with national policy and ensuring the project’s successful execution. Consequently, the team shifted to developing the final CASI-Plus prototype in REDCap, a platform already well established in Ukraine, even though it lacked certain desirable features identified during the requirement-gathering and design stages. The REDCap tool was hosted on PHC servers and was accessible as a web application to authorized users, meaning that internet connectivity at the sites was required for its use.

A total of 5 members of the PHC research team and 3 members of the University of Washington research team completed heuristic testing of the REDCap tool. Resulting improvements to CASI-Plus usability included (1) more consistent ways to advance between screens, with messages to inform clients about progress through the questionnaire; (2) improved instructions for questionnaire items with single versus multiple response options; (3) removal of duplicate items and content; and (4) improved page loading of images (Table 5).

**Table 5:** Illustrative examples of findings of the usability heuristic evaluation—step 2 of the Computer-Assisted Self-Interview–Plus formative research in wartime Ukraine (October 2023-May 2024).

Heuristic	Finding (severity)	Change to software
Consistency and standards—users should not have to wonder whether different words, situations, or actions mean the same thing. Follow platform conventions.	Inconsistent messages at the end of each section of the tool before moving on to the next section (medium)	Harmonized language showing progress through the sections of the tool
Error prevention—even better than good error messages is a careful design that prevents a problem from occurring in the first place. Either eliminate error-prone conditions or check for them and present users with a confirmation option before they commit to the action.	Question flow allows users to enter a partner name while also indicating that they do not have a partner (eg, enter 2 mutually exclusive states at once; medium)	Modified question flow
Recognition rather than recall—minimize the user’s memory load by making objects, actions, and options visible. The user should not have to remember information from one part of the dialogue to another. Instructions for use of the system should be visible or easily retrievable whenever appropriate.	For some questions, it is not obvious that multiple responses are allowed (low)	Added the description “Multiple answers allowed” to the questions with multiple response options
Aesthetic and minimalist design—dialogues should not contain information that is irrelevant or rarely needed. Every extra unit of information in a dialogue competes with the relevant units.	Confusing labeling and use of “show more” buttons in the section on methods of partner notification (high)	Changed style for “show more” buttons in REDCap^a^

^a^REDCap: Research Electronic Data Capture (Vanderbilt University).

### Step 3: Usability Walk-Throughs

HCWs found that navigating the main menu to register and edit client records (an unmodifiable aspect of REDCap) was not intuitive but was learnable with practice and that navigating the client-facing sections of the tool was straightforward. They requested hosting separate instances of the REDCap tool for each study site to protect the confidentiality of client records across sites and prevent inappropriate editing of records by personnel from the other study sites. They recommended that future, scaled implementation of CASI-Plus would allow for selective access permissions by site, a feature built into the original OpenSRP-based tool but not available within REDCap. Overall, the HCWs were enthusiastic about testing the tool in clinical practice.

Clients also found CASI-Plus easy to use and understand. One user indicated that the tool “significantly simplifies notifying the partner; stories provide considerable support and confidence” (client; male).

There were several points of feedback. One client noted that the terms used for different partner notification methods were difficult to understand but that the explanations clarified the terminology. Another noted that it might be difficult to recall one’s date of diagnosis. Client feedback did not require changes to the tool.

## Discussion

### Principal Findings

More interventions are needed to contain Ukraine’s HIV epidemic and facilitate the effectiveness of large-scale APS programs in other low-resource settings. The CASI-Plus concept of an mHealth tool that gives persons with HIV a convenient way to learn about HIV index testing and report their partners and that helps HCWs track completion of partner testing was acceptable to both clients and HCWs in Ukraine. Prioritized features included information about the benefits of HIV index testing, a nonjudgmental self-guided questionnaire to report partners, client stories, and bright images to accompany text. Two-way SMS text messaging between clients and HCWs was not considered feasible to integrate within the workflow of understaffed public health clinics. Our final tool design and software platform choice (REDCap) reflected national policies and the principle of avoiding extra burden to clients and health workers, resulting in higher intention to engage with the tool.

Our focus was on developing a tool that would be of generalized relevance outside of Ukraine and that would also work well within the challenging conditions of Ukraine’s wartime setting. This reflects the HCD design principle of “designing at the margins,” in which tools that can address widespread problems are then adapted for marginalized use cases through an HCD process with the target population [59,60]. In our case, final choices for CASI-Plus features and design reflected the constraints of Ukraine’s wartime setting but could be considered prudent choices for all contexts where health care resources are strained, workloads and time management are issues, and the target client population is stigmatized. The selection of the REDCap platform was driven by existing Ukraine Ministry of Health familiarity and capabilities. REDCap lacked several desired features that would have been possible with OpenSRP, such as hierarchical access permissions that would enable HCWs to view and edit the data only from their site while enabling scaled use across multiple sites and a data model supporting capture of record-level updates over time (rather than overwriting values through an editing process). Implementations of CASI-Plus in other settings might adapt the design but make a different technology choice.

This study contributes to the sparse literature on HCD methods for digital health interventions in war and humanitarian settings. The formative research phase of the CASI-Plus study demonstrated how simplified HCD methods adapted to the wartime context gathered rich input on the prioritized features and design of the CASI-Plus tool. There are few research studies or frameworks discussing HCD in mHealth projects in war or humanitarian settings. Existing published work has focused on tools for supporting the mental health of soldiers [61,62] or mHealth apps for health promotion among communities of refugees who have fled humanitarian crises [63-66]. Most existing work illustrates the role of the setting in shaping the problems and needs but not the specific ways in which the researchers adapted or shaped HCD methods based on a crisis context. For example, Beeman et al [65] described HCD methods applied to the design of menstrual health spaces for women living in a refugee settlement in Uganda. Their HCD methods involved standard steps of design research, rough prototyping, live prototyping, and piloting [65]. The resulting intervention design addressed the lack of privacy, tenuous access to disposable sanitary products, and other challenges facing women in the humanitarian setting. Another study of an mHealth app for Syrian refugees in Lebanon, a structured electronic health record with both client-facing features designed to improve health literacy and provider-facing features designed to support clinical decision-making, represents a cautionary tale in which design took place without participatory end-user input. The resulting tool failed to ease the workload of HCWs, and uptake was limited to approximately 20% of clinical consultations [67]. In another study, Bartlett et al [64] reported on an evaluation of an HCD process for a health care app for refugees. The evaluation findings noted power differentials in the design process and the need for diverse sampling techniques for client engagement so that design does not favor the perspectives of those with greater privilege [64].

Our formative study is unique in reporting on how we adapted mHealth HCD methods to a wartime context where the health system is under tremendous stress. Our study demonstrated several principles for successful HCD in a wartime context. First, we approached data collection so that it would be safe and feasible for both participants and study team members, all of whom were living through challenges of war, including displacement from their own homes; disrupted power supplies, including in winter; interrupted access to their offices, computers, and files; need to take shelter from bombs at a moment’s notice; and having family members and friends risking their lives on the front lines. Drawing on the HCD principle of empathy, we relied on in-country investigators to guide the timing and design of participatory formative research activities, first brainstorming possible activities and then consulting with HCWs at the sites to confirm whether the activities would be feasible, hear their concerns, and modify plans. Second, we created a detailed facilitator guide for the HCD workshop and added guidance to workshop slides to support facilitators in real time in guiding the conversation toward the research aims. Third, we scheduled activities during the day (eg, in the afternoons before daily facility curfew closure times) and during weeks when wartime conditions (eg, fewer bombings and power outages) permitted.

Adoption of mHealth tools is always complex from a system perspective, affecting tasks by individuals, how teams work together, and how teams function within broader systems (the micro, meso, and macro levels discussed by Melles et al [18]). The complexity of absorbing system changes during war is amplified. Ukraine is struggling to maintain its health care delivery system in the face of direct attacks on health care infrastructure, departure of many health workers, and surging public health disease burden. Priorities for the health care system include carrying through with reforms started before the war (eg, decentralization of health care management and enabling of private providers to receive state funding to deliver approved HIV service packages) and adaptations resulting from the war (eg, modifications to the electronic data system for the HIV program [called the Information System for Socially Significant Diseases] to enable remote use and tracking of patients receiving HIV care outside of Ukrainian borders). However, many adaptive responses in health care are still not formally recognized by the government, such as providers communicating with patients via messaging apps such as Viber or Telegram. In terms of CASI-Plus, our final design and prototype adapted to the existing APS workflows and formal regulations rather than introducing large changes that could be difficult to implement or sustain within understaffed public-sector clinics during wartime.

### Future Priorities and Research

Future priorities for CASI-Plus technology development include features to reduce workloads for the already overworked HCWs, such as integrations with other existing routine data systems to avoid double data entry, and improved tracking of named partners. Future efforts are warranted to explore the use of 2-way SMS text messaging at a time when Ukrainian health policy makers can codify formal regulations for this type of technology. The next step in research on CASI-Plus for Ukraine’s APS program is a randomized controlled trial of the effect of the REDCap-based CASI-Plus on the number of partners named and tested for HIV. CASI-Plus has the potential to complement other strategies being used in Ukraine to support HIV case finding in the wartime context. Previous studies have identified that APSs applied to social networks, particularly for persons who inject drugs, are effective for HIV case finding [33]. A recent study combined a social network approach to APSs with a machine learning algorithm applied to screening data from index clients to identify the networks most likely to yield new HIV cases [68]. Future research could study the use of CASI-Plus as part of social network testing and in conjunction with a prediction algorithm, as well as cultural adaptation of CASI-Plus in other countries.

### Strengths and Limitations

Strengths of this study were the adaptation of HCD methods to Ukraine’s wartime context, the inclusion of perspectives from 2 regions and both larger and smaller HIV service sites, and openness to HCW and client perspectives about the direction for CASI-Plus. Limitations of this study included the inability of HCWs and research team members to meet in person for creative co-design sessions; time pressure to complete the HCW workshops, where several participants had to drop out early when their worksites closed; and low engagement from rural, peripheral sites due to unfamiliarity with Zoom videoconferencing technology. Favorable opinions expressed about the CASI-Plus concept may reflect social desirability bias or a tendency to conform to the priorities of external funders because of the resources they provide [69]. Engaging participants from only 3 out of hundreds of primary health care sites may also have limited feature elicitation and utility across the spectrum of service delivery, particularly given the nuances in wartime settings. A limitation of pursuing CASI-Plus technology design during wartime was that national policies on technology platforms and patient electronic communications were less flexible than we anticipated when we conceptualized the study before the war, meaning that we were not able to implement 2-way SMS text messaging as part of the tool.

### Conclusion

In conclusion, the CASI-Plus mHealth tool is an acceptable intervention to support HIV testing targeted at people most at risk of HIV exposure, namely, the sexual and needle-sharing partners of people who have been newly diagnosed with HIV. It was possible to adapt HCD methods grounded in principles of empathy, iteration, and creative ideation to Ukraine’s wartime context using internet-based workshops with HCWs and individual interviews with clients. This work demonstrates the flexibility and value of HCD methods for adapting digital health tools in a wartime context.

## Appendix

Multimedia Appendix 1 Usability heuristic template for step 2 Computer-Assisted Self-Interview–Plus heuristic evaluation in wartime Ukraine (October 2023-May 2024).

Multimedia Appendix 2 Feedback on the Computer-Assisted Self-Interview–Plus proposed features and resulting design modifications based on the step 1 formative research in wartime Ukraine (May 2023-July 2023).

Multimedia Appendix 3 Prioritization of Computer-Assisted Self-Interview–Plus features by health care workers during step 1 formative workshops in wartime Ukraine (May 2023-July 2023).

## Abbreviations

APS: assisted partner service
CASI-Plus: Computer-Assisted Self-Interview–Plus
HCD: human-centered design
HCW: health care worker
mHealth: mobile health
PHC: Public Health Center
REDCap: Research Electronic Data Capture

## Data Availability

The datasets generated during and/or analyzed during this study are available from the corresponding author on reasonable request.
