# Supplementary material for: Human-Centered Design of an mHealth Tool for Optimizing HIV Index Testing in Wartime Ukraine: Formative Research Case Study
Source: JMIR Form Res. 2025 Jan 30;9:e66132. doi: 10.2196/66132 (PMC11826939; doi:10.2196/66132)
Supplement: Multimedia Appendix 1 [file formative_v9i1e66132_app1.docx]

**Supplemental Table 1: Usability heuristics template for step 2 CASI-Plus heuristic evaluation in wartime Ukraine (October 2023-May 2024)**

| **CASI-Plus Heuristic Evaluation** | | | | | |
| --- | --- | --- | --- | --- | --- |
| **Name:** | | | | | |
| **Date:** | | | | | |
| **Heuristic** (Source; N=Nielsen; K=Kientz) | **Section of System** | **Pass/Fail** | **Description of the problem** | **Severity** (Low, Mid, High) | **Design change recommendation** |
| **Visibility of system status (N)** The system should always keep users informed about what is going on, through appropriate feedback within reasonable time. | REDCap menu |  |  |  |  |
|  | Registration of index client |  |  |  |  |
|  | Why partner notification |  |  |  |  |
|  | Time window |  |  |  |  |
|  | Sex partners |  |  |  |  |
|  | Injection partners |  |  |  |  |
|  | Risks of notifying |  |  |  |  |
|  | Methods of notifying |  |  |  |  |
|  | Stories |  |  |  |  |
|  | Biological children |  |  |  |  |
|  | Feedback on CASI-Plus |  |  |  |  |
|  | IS SSD report |  |  |  |  |
|  | View and edit responses |  |  |  |  |
|  |  |  |  |  |  |
| **Match between system and the real world (N)** The system should speak the users' language, with words, phrases and concepts familiar to the user, rather than system-oriented terms. Follow real-world conventions, making information appear in a natural and logical order. | REDCap menu |  |  |  |  |
|  | Registration of index client |  |  |  |  |
|  | Why partner notification |  |  |  |  |
|  | Time window |  |  |  |  |
|  | Sex partners |  |  |  |  |
|  | Injection partners |  |  |  |  |
|  | Risks of notifying |  |  |  |  |
|  | Methods of notifying |  |  |  |  |
|  | Stories |  |  |  |  |
|  | Biological children |  |  |  |  |
|  | Feedback on CASI-Plus |  |  |  |  |
|  | IS SSD report |  |  |  |  |
|  | View and edit responses |  |  |  |  |
|  |  |  |  |  |  |
| **User control and freedom (N)** Users often choose system functions by mistake and will need a clearly marked "emergency exit" to leave the unwanted state without having to go through an extended dialogue. Support undo and redo. | REDCap menu |  |  |  |  |
|  | Registration of index client |  |  |  |  |
|  | Why partner notification |  |  |  |  |
|  | Time window |  |  |  |  |
|  | Sex partners |  |  |  |  |
|  | Injection partners |  |  |  |  |
|  | Risks of notifying |  |  |  |  |
|  | Methods of notifying |  |  |  |  |
|  | Stories |  |  |  |  |
|  | Biological children |  |  |  |  |
|  | Feedback on CASI-Plus |  |  |  |  |
|  | IS SSD report |  |  |  |  |
|  | View and edit responses |  |  |  |  |
|  |  |  |  |  |  |
| **Consistency and standards (N)** Users should not have to wonder whether different words, situations, or actions mean the same thing. Follow platform conventions. | REDCap menu |  |  |  |  |
|  | Registration of index client |  |  |  |  |
|  | Why partner notification |  |  |  |  |
|  | Time window |  |  |  |  |
|  | Sex partners |  |  |  |  |
|  | Injection partners |  |  |  |  |
|  | Risks of notifying |  |  |  |  |
|  | Methods of notifying |  |  |  |  |
|  | Stories |  |  |  |  |
|  | Biological children |  |  |  |  |
|  | Feedback on CASI-Plus |  |  |  |  |
|  | IS SSD report |  |  |  |  |
|  | View and edit responses |  |  |  |  |
|  |  |  |  |  |  |
| **Error prevention (N)** Even better than good error messages is a careful design which prevents a problem from occurring in the first place. Either eliminate error-prone conditions or check for them and present users with a confirmation option before they commit to the action. | REDCap menu |  |  |  |  |
|  | Registration of index client |  |  |  |  |
|  | Why partner notification |  |  |  |  |
|  | Time window |  |  |  |  |
|  | Sex partners |  |  |  |  |
|  | Injection partners |  |  |  |  |
|  | Risks of notifying |  |  |  |  |
|  | Methods of notifying |  |  |  |  |
|  | Stories |  |  |  |  |
|  | Biological children |  |  |  |  |
|  | Feedback on CASI-Plus |  |  |  |  |
|  | IS SSD report |  |  |  |  |
|  | View and edit responses |  |  |  |  |
|  |  |  |  |  |  |
| **Recognition rather than recall (N)** Minimize the user's memory load by making objects, actions, and options visible. The user should not have to remember information from one part of the dialogue to another. Instructions for use of the system should be visible or easily retrievable whenever appropriate. | REDCap menu |  |  |  |  |
|  | Registration of index client |  |  |  |  |
|  | Why partner notification |  |  |  |  |
|  | Time window |  |  |  |  |
|  | Sex partners |  |  |  |  |
|  | Injection partners |  |  |  |  |
|  | Risks of notifying |  |  |  |  |
|  | Methods of notifying |  |  |  |  |
|  | Stories |  |  |  |  |
|  | Biological children |  |  |  |  |
|  | Feedback on CASI-Plus |  |  |  |  |
|  | IS SSD report |  |  |  |  |
|  | View and edit responses |  |  |  |  |
|  |  |  |  |  |  |
| **Flexibility and efficiency of use (N)** Accelerators — unseen by the novice user — may often speed up the interaction for the expert user such that the system can cater to both inexperienced and experienced users. Allow users to tailor frequent actions. | REDCap menu |  |  |  |  |
|  | Registration of index client |  |  |  |  |
|  | Why partner notification |  |  |  |  |
|  | Time window |  |  |  |  |
|  | Sex partners |  |  |  |  |
|  | Injection partners |  |  |  |  |
|  | Risks of notifying |  |  |  |  |
|  | Methods of notifying |  |  |  |  |
|  | Stories |  |  |  |  |
|  | Biological children |  |  |  |  |
|  | Feedback on CASI-Plus |  |  |  |  |
|  | IS SSD report |  |  |  |  |
|  | View and edit responses |  |  |  |  |
|  |  |  |  |  |  |
| **Aesthetic and minimalist design (N)** Dialogues should not contain information which is irrelevant or rarely needed. Every extra unit of information in a dialogue competes with the relevant units of information and diminishes their relative visibility. | REDCap menu |  |  |  |  |
|  | Registration of index client |  |  |  |  |
|  | Why partner notification |  |  |  |  |
|  | Time window |  |  |  |  |
|  | Sex partners |  |  |  |  |
|  | Injection partners |  |  |  |  |
|  | Risks of notifying |  |  |  |  |
|  | Methods of notifying |  |  |  |  |
|  | Stories |  |  |  |  |
|  | Biological children |  |  |  |  |
|  | Feedback on CASI-Plus |  |  |  |  |
|  | IS SSD report |  |  |  |  |
|  | View and edit responses |  |  |  |  |
|  |  |  |  |  |  |
| **Help users recognize, diagnose, and recover from errors (N)** Error messages should be expressed in plain language (no codes), precisely indicate the problem, and constructively suggest a solution. | REDCap menu |  |  |  |  |
|  | Registration of index client |  |  |  |  |
|  | Why partner notification |  |  |  |  |
|  | Time window |  |  |  |  |
|  | Sex partners |  |  |  |  |
|  | Injection partners |  |  |  |  |
|  | Risks of notifying |  |  |  |  |
|  | Methods of notifying |  |  |  |  |
|  | Stories |  |  |  |  |
|  | Biological children |  |  |  |  |
|  | Feedback on CASI-Plus |  |  |  |  |
|  | IS SSD report |  |  |  |  |
|  | View and edit responses |  |  |  |  |
|  |  |  |  |  |  |
| **Help and documentation (N)** Even though it is better if the system can be used without documentation, it may be necessary to provide help and documentation. Any such information should be easy to search, focused on the user's task, list concrete steps to be carried out, and not be too large. | REDCap menu |  |  |  |  |
|  | Registration of index client |  |  |  |  |
|  | Why partner notification |  |  |  |  |
|  | Time window |  |  |  |  |
|  | Sex partners |  |  |  |  |
|  | Injection partners |  |  |  |  |
|  | Risks of notifying |  |  |  |  |
|  | Methods of notifying |  |  |  |  |
|  | Stories |  |  |  |  |
|  | Biological children |  |  |  |  |
|  | Feedback on CASI-Plus |  |  |  |  |
|  | IS SSD report |  |  |  |  |
|  | View and edit responses |  |  |  |  |
|  |  |  |  |  |  |
| **Appropriate functionality (K)** The technology should function effectively in the user’s environment by being easy to use and integrate into their daily routine. | REDCap menu |  |  |  |  |
|  | Registration of index client |  |  |  |  |
|  | Why partner notification |  |  |  |  |
|  | Time window |  |  |  |  |
|  | Sex partners |  |  |  |  |
|  | Injection partners |  |  |  |  |
|  | Risks of notifying |  |  |  |  |
|  | Methods of notifying |  |  |  |  |
|  | Stories |  |  |  |  |
|  | Biological children |  |  |  |  |
|  | Feedback on CASI-Plus |  |  |  |  |
|  | IS SSD report |  |  |  |  |
|  | View and edit responses |  |  |  |  |
|  |  |  |  |  |  |
| **Not irritating (K)** The technology should not irritate or embarrass the user, even after using the product repeatedly and regularly over a long period of time. | REDCap menu |  |  |  |  |
|  | Registration of index client |  |  |  |  |
|  | Why partner notification |  |  |  |  |
|  | Time window |  |  |  |  |
|  | Sex partners |  |  |  |  |
|  | Injection partners |  |  |  |  |
|  | Risks of notifying |  |  |  |  |
|  | Methods of notifying |  |  |  |  |
|  | Stories |  |  |  |  |
|  | Biological children |  |  |  |  |
|  | Feedback on CASI-Plus |  |  |  |  |
|  | IS SSD report |  |  |  |  |
|  | View and edit responses |  |  |  |  |
|  |  |  |  |  |  |
| **Protects privacy (K)** The system allows users to keep personal information private. Users can control what, when, to whom, how, and how much information is made public. Any public information is kept abstract. | REDCap menu |  |  |  |  |
|  | Registration of index client |  |  |  |  |
|  | Why partner notification |  |  |  |  |
|  | Time window |  |  |  |  |
|  | Sex partners |  |  |  |  |
|  | Injection partners |  |  |  |  |
|  | Risks of notifying |  |  |  |  |
|  | Methods of notifying |  |  |  |  |
|  | Stories |  |  |  |  |
|  | Biological children |  |  |  |  |
|  | Feedback on CASI-Plus |  |  |  |  |
|  | IS SSD report |  |  |  |  |
|  | View and edit responses |  |  |  |  |
|  |  |  |  |  |  |
| **Aesthetically appealing (K)** The design of the technology is attractive and appealing and adheres to basic usability standards. The design captures and sustains the user’s interest, enhances user engagement with the technology, and also adds to the credibility and usability of the product. | REDCap menu |  |  |  |  |
|  | Registration of index client |  |  |  |  |
|  | Why partner notification |  |  |  |  |
|  | Time window |  |  |  |  |
|  | Sex partners |  |  |  |  |
|  | Injection partners |  |  |  |  |
|  | Risks of notifying |  |  |  |  |
|  | Methods of notifying |  |  |  |  |
|  | Stories |  |  |  |  |
|  | Biological children |  |  |  |  |
|  | Feedback on CASI-Plus |  |  |  |  |
|  | IS SSD report |  |  |  |  |
|  | View and edit responses |  |  |  |  |
|  |  |  |  |  |  |
| **Accuracy of information (K)** The technology should not inaccurately record or misrepresent the user’s responses. The technology should allow users to edit data records and/or manually input additional data. | REDCap menu |  |  |  |  |
|  | Registration of index client |  |  |  |  |
|  | Why partner notification |  |  |  |  |
|  | Time window |  |  |  |  |
|  | Sex partners |  |  |  |  |
|  | Injection partners |  |  |  |  |
|  | Risks of notifying |  |  |  |  |
|  | Methods of notifying |  |  |  |  |
|  | Stories |  |  |  |  |
|  | Biological children |  |  |  |  |
|  | Feedback on CASI-Plus |  |  |  |  |
|  | IS SSD report |  |  |  |  |
|  | View and edit responses |  |  |  |  |
|  |  |  |  |  |  |
| **Educate users (K)** The technology should engage users in an active process whereby they learn information and gain skills relevant to their goals, particularly skills that would enable them to continue to progress towards goals even in the absence of the technology. | REDCap menu |  |  |  |  |
|  | Registration of index client |  |  |  |  |
|  | Why partner notification |  |  |  |  |
|  | Time window |  |  |  |  |
|  | Sex partners |  |  |  |  |
|  | Injection partners |  |  |  |  |
|  | Risks of notifying |  |  |  |  |
|  | Methods of notifying |  |  |  |  |
|  | Stories |  |  |  |  |
|  | Biological children |  |  |  |  |
|  | Feedback on CASI-Plus |  |  |  |  |
|  | IS SSD report |  |  |  |  |
|  | View and edit responses |  |  |  |  |
