# Supplementary material for: Human-Centered Design of an mHealth Tool for Optimizing HIV Index Testing in Wartime Ukraine: Formative Research Case Study
Source: JMIR Form Res. 2025 Jan 30;9:e66132. doi: 10.2196/66132 (PMC11826939; doi:10.2196/66132)
Supplement: Multimedia Appendix 2 [file formative_v9i1e66132_app2.doc]

**Supplemental Table 2: Feedback on CASI-Plus proposed features and resulting design modifications based on step 1 formative research in wartime Ukraine (May-July 2023)**

| **Client journey step** | **CASI-Plus proposed feature** | **Feedback from HCW and clients** | **Design modification** |
| --- | --- | --- | --- |
| **Initial visit** | | | |
| **Introduce concept of index testing** | Standardized introductory health education content on purpose of index testing | Appreciate how tool reinforces key education messages and reduces pressure on HCW to provide education (HCW) Need to normalize HIV and provide supportive messaging to clients before discussing APS (e.g. praise for care-seeking behavior, emphasize availability and effectiveness of ART to protect own health and protect partners [U=U]) (clients) | Modified health education messages to validate client's positive health seeking behaviors and self-care, emphasize effectiveness of treatment and U=U messages |
| **Invitation to participate in the index testing program** | Client stories (role modeling) | Some clients will not take time to read, others may like (HCW and clients) Want stories to be more realistic (HCW and clients) | Rewrote stories using client profiles provided by health workers and themes from in-depth client interviews |
| **Eliciting information about partners** | Standard partner elicitation questionnaire with non-judgmental language | Tool could make it easier for some to disclose partners (HCW and clients) Questionnaire was generally clear but need to reword questions about client HIV testing history (clients) | Revised questions on client testing history to inform exposure window of interest |
| **Screening for risk of violence** | Standard questionnaire to ask about IPV risk for named partners | Concern that question on risk of sexual violence is "too personal" (clients) | Did not modify questions due to need to be consistent with IS SSD |
| **Choice of partner notification method** | Explanations of each type of method; Client stories that illustrate different methods | Clear explanations (HCW and clients) Could reduce HCW workload (HCW) | No change |
| **Verification of information and data entry** | HCW ability to review client responses together with client, and edit if needed; Summary view of partner data captured in CASI-Plus (to enable rapid data entry to IS-SSD) | Necessary step to validate data (HCW) Concern about burden of entering data captured in CASI-Plus to IS- SSD (HCW) | No change |
| **Planning your next visit** | Not covered in CASI-Plus | NA | NA |
| **Follow up case management** | | | |
| **Reminders about notification** | Automated SMS reminders with link to automated follow-up questionnaire | Concern about workflow changes and extra workload, even if SMS messages were automated (HCW) Concern about needing an exception to current policy on use of clients' personal data (HCW) Convenient (clients) Use of encrypted messages would alleviate privacy concerns (clients) | Removed SMS messaging Added follow up form for CASI-Plus use within clinic during follow up client visits |
| **Contacting partner** | Automated follow-up questionnaire checks on completion of partner notification Automated follow-up questionnaire invites switch to assisted partner notification if facing challenges with self-notification | Could be helpful to client case management, especially if data automatically integrated with IS SSD (HCW) | Follow-up surveys collected within clinic on tablets, when clients return for follow-up visits, rather than remotely by SMS link CASI-Plus interface allows HCW to lookup existing clients registered in CASI-Plus and select for initial vs. follow-up visits |
| **Reporting back on notification and testing of named partners** | Automated follow-up questionnaire invites report-back on disposition of named partners | Could be helpful to client case management, especially if data automatically integrated with IS SSD (HCW) |
| **Partner case closure** | View of open cases, ability to close cases if testing completed or partner not reachable | Dashboard should require minimal duplicate data entry by HCW (HCW) | Dashboard available but focused on displaying information needed for individual case management rather than KPIs Dashboard relies on information reported by clients from questionnaires, not data entry by HCW |
| **Eliciting new partners** | Not anticipated as part of initial design | Clients often report new partners at a later visit, after building trust (HCW) | Automated follow-up questionnaire invites naming of new partners |
| **Viewing reports** | Performance management reports (showing key performance indicators) | Frontline HCW do not use data summaries (HCW) | Dashboard available but focused on displaying information needed for individual case management rather than KPIs |
