# Supplementary material for: Human-Centered Design of an mHealth Tool for Optimizing HIV Index Testing in Wartime Ukraine: Formative Research Case Study
Source: JMIR Form Res. 2025 Jan 30;9:e66132. doi: 10.2196/66132 (PMC11826939; doi:10.2196/66132)
Supplement: Multimedia Appendix 3 [file formative_v9i1e66132_app3.docx]

**Supplemental Table 3: Prioritization of CASI-Plus features by healthcare workers (HCW) during step 1 formative workshops in wartime Ukraine (May – July 2023)**

|  | Chernihiv Oblast Center for HIV/AIDS and Hepatitis | | | Chernihiv peripheral primary care sites | | | Dnipropetrovsk Regional Medical Center of Socially Significant Diseases | | |
| --- | --- | --- | --- | --- | --- | --- | --- | --- | --- |
| Ideas | R1 | R2 | R3 | R1 | R2 | R3 | R1 | R2 | R3 |
| HCW reads intro script that describes APS | 0 | 0 | 0 |  |  |  |  |  |  |
| Client reads intro script that describes APS | 6 | 6 | 0 |  |  |  |  |  |  |
| HCW locks tablet using security cable |  |  |  | 3 | 2 | 0 | 2 | 2 | 2 |
| Client enters partner named or nickname and other details | 3 | 1 | 1 | 3 | 4 | 4 | 3 | 2 | 3 |
| Client selects how each partner should be notified | 2 | 6 | 0 | 2 | 2 | 4 | 3 | 4 | 2 |
| Client stories illustrate how APS works | 2 | 1 | 1 | 0 | 0 | 0 | 0 | 2 | 2 |
| HCW reviews responses with clients and edits as needed | 2 | 1 | 1 | 1 | 2 | 1 | 0 | 4 | 2 |
| Sends text message to initiate follow up | 2 | 2 | 0 | 0 | 0 | 0 |  |  |  |
| HCW views information on laptop | 1 | 2 | 1 | 3 | 3 | 3 | 0 | 0 | 0 |
| Summary view of data to support data entry to IS SSD | 0 | 0 | 0 | 0 | 0 | 0 | 4 | 2 | 4 |
| Use CASI-Plus for two-way texting | 0 | 0 | 2 | 0 | 0 | 0 | 0 | 0 | 1 |
| CASI-Plus automatically sends reminder messages to clients | 0 | 0 | 4 | 0 | 0 | 0 | 0 | 0 | 0 |
| Displays line list of partners with phone numbers for easy contacting | 1 | 1 | 1 | 0 | 0 | 0 |  |  |  |
| Displays line list of open case files | 0 | 0 | 1 | 0 | 0 | 0 |  |  |  |
| Displays line list of closed case files to access later | 0 | 0 | 0 | 0 | 0 | 0 |  |  |  |
| Dashboard shows lists of clients and partners with their testing status |  |  |  | 0 | 0 | 0 | 6 | 4 | 5 |
| Dashboard shows data summaries and performance indicators | 0 | 0 | 2 | 0 | 0 | 0 |  |  |  |
| Export Excel or CSV files for data analysis |  |  |  | 0 | 0 | 0 | 3 | 1 | 0 |

|  | Indicates the idea was not part of voting at the site (idea lists differed by workshop). |
| --- | --- |
